# The contribution of edible cricket flour to quality parameters and sensory characteristics of wheat bread

**DOI:** 10.1002/fsn3.3024

**Published:** 2022-08-09

**Authors:** Elena Bartkiene, Vytaute Starkute, Konstantinas Katuskevicius, Neringa Laukyte, Markas Fomkinas, Edikas Vysniauskas, Paulina Kasciukaityte, Emilis Radvilavicius, Skaiste Rokaite, Domantas Medonas, Emilija Valantinaviciute, Ernestas Mockus, Egle Zokaityte

**Affiliations:** ^1^ Department of Food Safety and Quality Lithuanian University of Health Sciences Kaunas Lithuania; ^2^ Institute of Animal Rearing Technologies Lithuanian University of Health Sciences Kaunas Lithuania

**Keywords:** cricket flour, edible insects, sensory properties, volatile compounds, wheat bread

## Abstract

This study evaluated the influence of edible cricket flour (ECF) on the quality parameters and sensory characteristics of wheat bread (WB), including the formation of volatile compounds (VC) and their relationship with emotions (EM) induced for consumers. ECF reduced dough pH, redness, and yellowness. At 5%, ECF increased the porosity of WB (by 7.87%). The quantity of ECF significantly affected WB's specific volume (*p* = .030), porosity (*p* = .0001), shape coefficient (*p* = .0001), and mass loss (*p* = .023). All WB with ECF had a more intense color and additive odor. Bread samples with 10% and 15% ECF had more intense overall, additive, acidity, and bitterness flavors. However, all WB had similar overall acceptability (OA) and no correlations were found between OA and VC. The EM “happy” and “sad” were expressed more intensely for WB with 15% ECF, and significant correlations were established between the EM “happy” and separate VC. The main VC in WB were ethanol; 1‐butanol and 3‐methyl; 1‐hexanol; estragole; and hexanoic acid. Finally, 5% ECF could be incorporated into the main WB formula without having a negative impact on bread quality. Also, ECF influences VC formation, and separate VC could be related to emotions induced for consumers.

## Graphical abstract

This study evaluated the influence of edible cricket flour (ECF) on the quality parameters and sensory characteristics of wheat bread (WB), including the formation of volatile compounds (VC) and their relationship with emotions (EM) induced for consumers. ECF influences VC formation, and separate VC could be related to emotions induced for consumers.

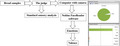

## 1 INTRODUCTION

The world's population is expected to reach 9.7 billion by 2050, increasing the demand for high biological value protein produced in a sustainable manner (United Nations, 2015). For this reason, the food industry has started to focus on sustainable protein sources, and insects have become a potential stock for the food industry. Insects are a traditional food in many countries (van Huis et al., 2013). Despite the fact that the European Union (EU) brought into force Regulation (EU), 2015/2283, recognizing insects as novel foods, there is no tradition of including insects in the diet in Western and Northern countries (Regulation (EU), 2015). The major obstacles to the low consumption of whole insects as food in these countries are identified as neophobia, disgust, and nonacceptance (Caparros Megido et al., 2016). However, the consumption of edible insects as ingredients in traditional food has become more and more popular (Cappelli et al., 2020; De Smet et al., 2019; González et al., 2019; Scholliers et al., 2019). Nowadays, there are many food products and beverages on the market (e.g., cinnamon choco‐chip seasoned roasted crickets; mini‐kickers set: savory flavored roasted cricket snacks; and crickets by the pound chocolate coffee), which are based on insects and (or) include edible insects as ingredient.

Wheat bread is the most popular food product around the world, and its enrichment with edible insect flour, as an ingredient, could be a good solution to improve the population's diet with high‐value protein. In addition, taking into consideration that bread is a staple food, this product must be well balanced from a nutritional point of view (Vatankhah et al., 2017), and bread enrichment with valuable ingredients can assist to reduce the challenge of malnutrition (Chinma et al., 2020). However, the incorporation of edible cricket flour into the main bread formula can lead to various changes—desirable (improved nutritional value) and undesirable (lower acceptability, lower porosity, etc.). Also, edible cricket flour can lead to changes in the bread's volatile compound (VC) profile because of the high quality of proteins that are involved in VC formation reactions during the bread making (e.g., fermentation) and baking processes. Odor is a very important sensory characteristic, which depends on the overall acceptability of the product, as well as emotions induced for consumers.

The aim of this study was to evaluate the influence of edible cricket flour on the quality parameters and sensory characteristics of wheat bread, including the formation of VC and their relationship with emotions induced for consumers.

## 2 MATERIALS AND METHODS

### 2.1 Materials used for bread preparation

Wheat flour (type 812C, falling number 315 s, gluten 30%, and ash 0.74%) obtained from Kauno Grudai Ltd. mill (Kaunas, Lithuania) was used for the wheat bread (WB) preparation experiment. The WB samples were prepared without and with the addition of edible cricket flour (5%, 10%, and 15%). Edible cricket flour was provided by Bugsandus Ltd. (Vilnius, Lithuania). Cricket flour composition is as follows: protein 62.6%, fat 26.5%, ash 3.8%, moisture 2.0%, saturated fatty acids 8.7%, total carbohydrates 5.1%, sugars <0.6%, and salt (sodium *×* 2.5) 0.69%.

### 2.2 Bread preparation

The WB formula consisted of 1 kg of wheat flour, 1.5% salt, 2% fresh compressed yeast, and 56% water (control bread). Control WB samples were prepared without the addition of edible cricket flour. The dough was mixed for 3 min at a low speed and then for 7 min at a high‐speed regime in a dough mixer (KitchenAid Artisan, Ohio, USA). Then, the dough was left at 22 *±* 2*°*C for 12 min relaxation. Next, the dough was shaped into 350 g loaves, formed, and proofed at 30 *±* 2*°*C and 80% relative humidity for 60 min. The bread was baked in a deck oven (EKA, Borgoricco PD, Italy) at 220*°*C for 25 min.

### 2.3 Evaluation of bread quality parameters

After 12 h of cooling at 22 *±* 2*°*C, WB samples were subjected to analysis of specific volume, crumb porosity, shape coefficient, mass loss after baking, crust and crumb color coordinates, sensory characteristics and overall acceptability, emotions induced for judges by the bread, and VC profile.

Bread volume was established by the AACC method (AACC, 2003), and the specific volume was calculated as the ratio of volume to weight. Bread crumb porosity was evaluated by LST method 1442:(1996) (LST, 1996). The bread shape coefficient was calculated as the ratio of bread slice width to height (in mm). Mass loss after baking was calculated as a percentage by measuring loaf dough mass before baking and after baking. Crust and crumb color parameters were evaluated using a CIE L*a*b* system (CromaMeter CR‐400, Konica Minolta, Japan; McGuire, 1992). Sensory characteristics and overall acceptability of the slices of bread were carried out by 10 trained judges according to ISO method 8586‐1 (ISO, 2012) using a 140 mm hedonic line scale ranging from 140 (extremely like) to 0 (extremely dislike).

**FIGURE 1:**
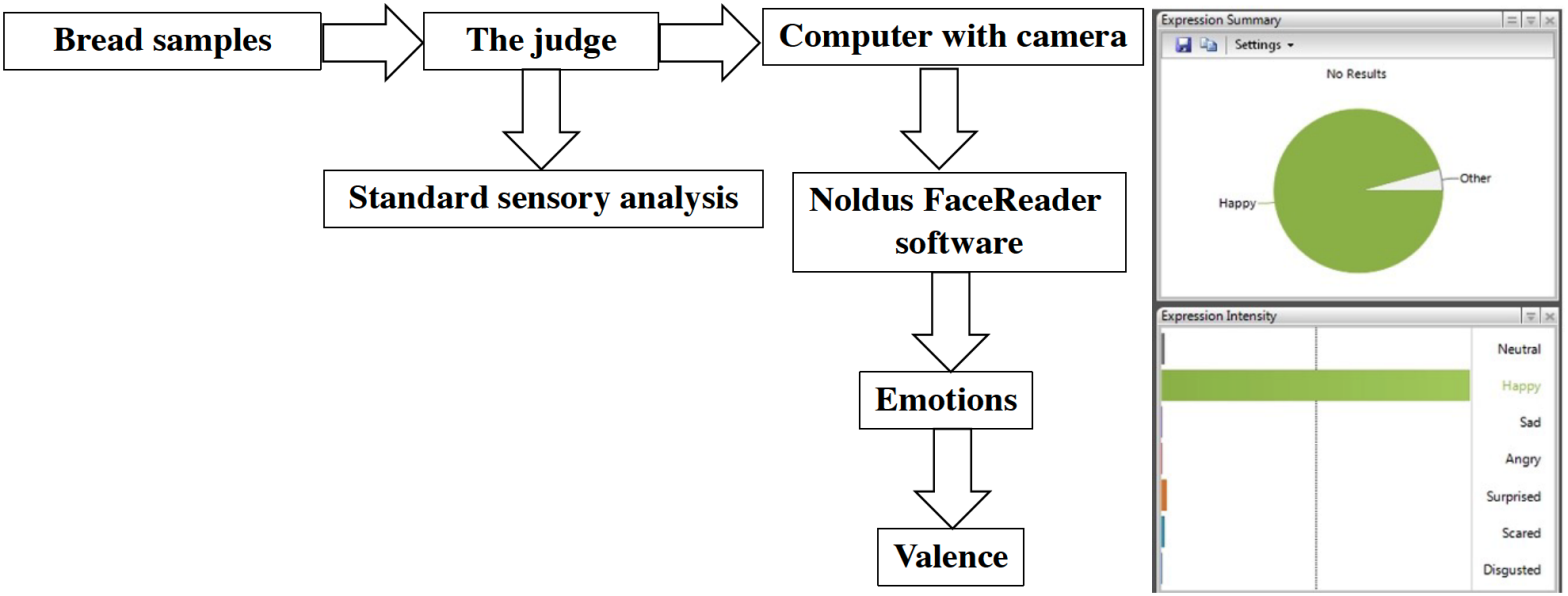
Analysis of the emotions induced by the bread using FaceReader software (Noldus Information Technology, Wageningen, the Netherlands), and further scoring the eight emotion patterns: neutral, happy, sad, angry, surprised, scared, disgusted, and contempt.

The slices of bread also were tested by applying FaceReader 8.0 software (Noldus Information Technology, Wageningen, The Netherlands; Figure 1), with a scoring scale of eight emotion patterns (neutral, happy, sad, angry, surprised, scared, disgusted, and contempt). The whole procedure is described in detail by Bartkiene, Jomantaite, et al. (2021a) (Figure 1). For statistical analysis, the maximum values of the facial expression patterns of the respective sections were used.

The VC of the bread samples were analyzed by gas chromatography–mass spectrometry (GC–MS) as described by Bartkiene, Mockus, et al. (2021b). A solid‐phase microextraction (SPME) device with Stableflex™ fiber coated with a 50 μm PDMS‐DVB‐Carboxen™ layer (Supelco, USA) was used for analysis. A whole slice of bread was weighed and blended with aqueous sodium chloride solution (30% w/v) in a ratio of 1 g of bread to 3 ml of NaCl solution. For headspace extraction, 8 g of prepared sample was transferred to the 20 ml extraction vial, sealed with a polytetrafluoroethylene septum, and thermostatted at 60°C for 15 min before exposing the fiber in the headspace. The fiber was exposed to the headspace of the vial for 10 min and desorbed in an injector liner for 2 min (splitless injection mode). Prepared samples were analyzed with a GCMS‐QP2010 (Shimadzu, Japan) gas chromatograph and mass spectrometer. The following conditions were used for analysis: injector temperature 250°C, ion source temperature 220°C, and interface temperature 260°C. Helium was used as carrier gas at 0.95 ml min^−1^ flow rate. A Stabilwax‐DA capillary column (0.25 mm ID, 0.25 μm film thickness, and 30 m length [Restek, USA]) was used for analysis. The temperature gradient was programmed from the start at 40°C (3 min hold) to 220°C (6°C min^−1^) up to 250°C (10°C min^−1^; 6 min hold). The VC were identified according to mass spectrum libraries (NIST11, NIST11S, and FFNSC2).

### 2.4 Statistical analysis

The results were expressed as the mean values (for dough and bread samples *n* = 3; and for bread sensory characteristics, overall acceptability, and emotions induced for trained judges *n* = 10) ± standard error (SE). In order to evaluate the effects of different quantities of edible cricket flour on WB quality parameters, data were analyzed by one‐way ANOVA and Tukey HSD tests as post hoc tests (statistical program R 3.2.1). Also, Pearson correlations were calculated between various parameters, as well as between the separate VC and overall acceptability of the bread, and between VC and the emotion “happy” induced for judges. The results were recognized as statistically significant at *p* ≤ .05.

## 3 RESULTS AND DISCUSSION

### 3.1 Influence of edible cricket flour on dough acidity parameters, color characteristics, and hardness

Dough acidity parameters and color characteristics are given in Table 1. The lowest pH and TTA were established for the doughs prepared with 10% edible cricket flour (13.3% and 52.0% lower, respectively, in comparison with control doughs). A very strong positive correlation was found between dough pH and TTA (*r* = .8510, *p* = .0001). The positive correlation between dough acidity parameters (pH and TTA) can be explained by proteolysis, which can lead to the formation of alkaline compounds and the neutralization of dissociated acids in dough. Also, volatilization of small molecular acids during the process is possible. As well as that, the incorporation of proteinaceous ingredients (e.g., edible cricket flour) in the main bread formula can lead to more free amino acids being released into the medium, and this can contribute to more effective yeast growth. In addition, amino acids take part in the Maillard reaction and can lead to a more intense odor, as well as color, of the product.

**TABLE 1:** Dough acidity parameters and color characteristics

Dough samples	Acidity parameters		Color characteristics		
	pH	TTA, °N	L*	a*	b*
Control	5.62 ± 0.10^c^	20.1 ± 1.3^c^	78.6 ± 2.4^b^	1.53 ± 0.05^a^	21.8 ± 0.4^a^
Cr‐5%	5.32 ± 0.07^b^	11.2 ± 0.3^b^	79.7 ± 0.5^b^	2.34 ± 0.30^b^	23.5 ± 0.4^b^
Cr‐10%	4.93 ± 0.11^a^	9.65 ± 0.50^a^	77.6 ± 2.0^a^	3.33 ± 0.15^c^	24.8 ± 0.2^c^
Cr‐15%	5.32 ± 0.04^b^	11.2 ± 0.5^b^	79.7 ± 0.5^b^	2.34 ± 0.15^b^	23.5 ± 0.5^b^

Influence of analyzed factor (different quantities of cricket flour) on dough parameters		
Dough parameters	*F*	*p*
pH	34.741	.0001
TTA, °N	119.486	.0001
L*	1.193	.372
a*	47.363	.0001
b*	29.770	.0001

*Note*: Data expressed as mean values (*n* = 3) ± standard deviation (SD). The influence of analyzed factor (different quantities of cricket flour) on dough parameters is significant when *p* ≤ .05. The control dough was produced without edible cricket flour; Cr‐5%, Cr‐10%, and Cr‐15%—dough was produced with 5%, 10%, and 15%, respectively, edible cricket flour. ^a–c^Mean values within a row with different letters are significantly different (*p* ≤ .05).

Abbreviations: L* lightness; a* redness or −a* greenness; b* yellowness or −b* blueness; NBS, National Bureau of Standards units.

Comparing dough color characteristics, the addition of edible cricket flour increased dough redness (a*) and yellowness (b*) coordinates (on average, by 74.5% and 9.77%, respectively, in comparison with control doughs), and the doughs prepared with 10% edible cricket flour had the highest a* and b* (3.33 and 24.8 NBS, respectively). Also, doughs prepared with 10% edible cricket flour had the lowest lightness (L*; on average, 2.2% lower than in control doughs and doughs prepared with 5% and 15% edible cricket flour).

Very strong negative correlations were found between the dough acidity parameters (pH and TTA) and a* and b* color characteristics (between pH and TTA and a*: *r* = *−*.8920, *p* = .0001; and *r* = *−*.8110, *p* = .001, respectively; and between pH and TTA and b*: *r* = *−*.8500, *p* = .0001; and *r* = *−*.8490, *p* = .0001, respectively). However, the correlations between dough acidity parameters and L* were not significant. The influence of the analyzed factor (different quantities of edible cricket flour) on dough pH, TTA, and a* and b* color characteristics was significant (*p* = .0001); however, the quantity of edible cricket flour incorporated was not a significant factor in dough L* color coordinate (Table 1).

### 3.2 Influence of edible cricket flour on wheat bread specific volume, porosity, shape coefficient, mass loss after baking, and crust and crumb color characteristics

The main quality characteristics of WB, prepared without and with the different quantities of edible cricket flour (specific volume, porosity, shape coefficient, mass loss after baking, and crust and crumb color characteristics), are given in Table 2. Comparing bread's specific volume and porosity, the addition of 5% edible cricket flour did not have a significant effect on bread's specific volume in comparison with slices of control bread but slices of bread with 5% edible cricket flour had 7.87% higher porosity than the control. However, the addition of 10% and 15% edible cricket flour to the main bread formula reduced bread's specific volume and porosity (on average, by 6.00% and 13.0%, respectively, compared to slices of control bread). Cappelli et al. (2020) reported that bread volume decreases as the percentage of cricket substitution increases. These changes can be related to the dilution of gluten and a weakening of its network, which leads to a lower gas retention capacity and causes inferior rheological performance of the dough (DeFloor et al., 1993; González et al., 2019). However, adding 5% edible cricket flour did not have a significant effect on the bread's specific volume and porosity; this can be explained by the cricket flour being a good source of additional amino acids for yeast growth. The results for bread crust color coordinates confirm this explanation because the crust of bread slices with 5% edible cricket flour showed similar color characteristics to slices of control bread. This can be explained by the fact that the additional amino acids from cricket flour were not involved in the Maillard reaction but were consumed by yeasts during dough fermentation. Also, a strong positive correlation was found between bread's specific volume and porosity (*r* = .780, *p* = .003); strong and moderate positive correlations were established between bread's specific volume and porosity and bread mass loss after baking (*r* = .631, *p* = .028 and *r* = .576, *p* = .050, respectively). These findings can be explained by more intensive water migration during the baking process from bread that is more porous and has a higher specific volume, which leads to loaves with a lower mass. Bread samples containing edible cricket flour had a higher shape coefficient in comparison with control bread samples, the highest being found in slices of bread with 5% edible cricket flour (2.83). The analyzed factor (different quantities of cricket flour) had a significant influence on WB specific volume (*p* = .030), porosity (*p* = .0001), shape coefficient (*p* = .0001), and mass loss after baking (*p* = .023; Table 2).

**TABLE 2:** Bread specific volume, porosity, shape coefficient, mass loss after baking, color characteristics of the bread crust and crumb, and bread crumb images

Bread samples	Specific volume, cm^3^ g^−1^	Porosity, %	Shape coefficient	Mass loss after baking, %
Control	2.12 ± 0.08^c^	62.3 ± 1.5^c^	1.89 ± 0.10^a^	14.7 ± 3.7^b^
Cr‐5%	2.52 ± 0.33^c^	67.2 ± 1.2^d^	2.83 ± 0.06^d^	15.5 ± 0.8^b,c^
Cr‐10%	2.00 ± 0.35^a^	57.8 ± 0.1^b^	2.25 ± 0.02^b^	9.50 ± 0.2^a^
Cr‐15%	2.00 ± 0.12^a^	52.5 ± 1.7^a^	2.52 ± 0.12^c^	12.9 ± 0.9^b^

Bread samples	Crust			Crumb		
	L*	a*	b*	L*	a*	b*
Control	52.8 ± 3.4^a^	9.87 ± 0.33^b^	25.4 ± 0.4^b^	67.9 ± 0.9^d^	4.21 ± 0.12^b^	20.6 ± 0.3^a^
Cr‐5%	47.5 ± 0.8^a^	10.0 ± 0.21^b^	18.7 ± 0.1^a^	64.6 ± 0.5^c^	3.61 ± 0.25^a^	23.8 ± 2.1^a^
Cr‐10%	57.4 ± 2.4^c^	8.55 ± 0.27^a^	26.0 ± 1.8^b^	62.7 ± 0.6^b^	4.32 ± 0.07^b^	26.5 ± 0.3^b^
Cr‐15%	59.2 ± 1.3^c^	8.73 ± 0.12^a^	29.5 ± 0.4^c^	56.6 ± 1.2^a^	6.01 ± 0.37^c^	27.5 ± 0.7^c^

Influence of analyzed factor (different quantities of cricket flour) on bread parameters			
Bread parameters		*F*	*p*
Specific volume, cm^3^ g^−1^		5.050	.030
Porosity, %		71.751	.0001
Shape coefficient		67.447	.0001
Mass loss after baking, %		5.615	.023
Crust	L*	16.710	.001
	a*	28.326	.0001
	b*	68.415	.0001
Crumb	L*	94.559	.0001
	a*	58.218	.0001
	b*	22.567	.0001

Bread crumb images	
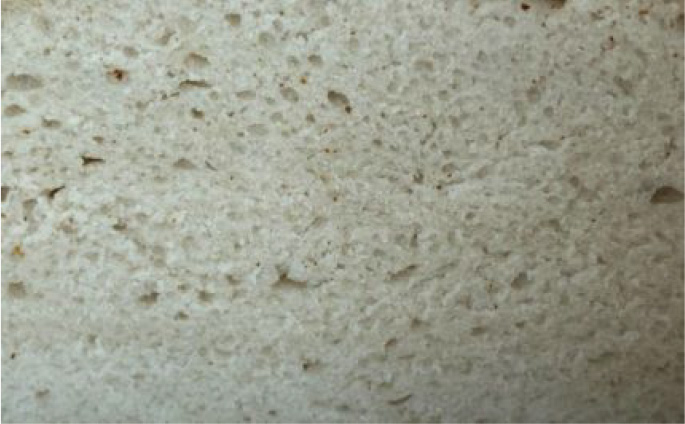	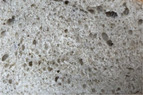
Control	Cr‐5%
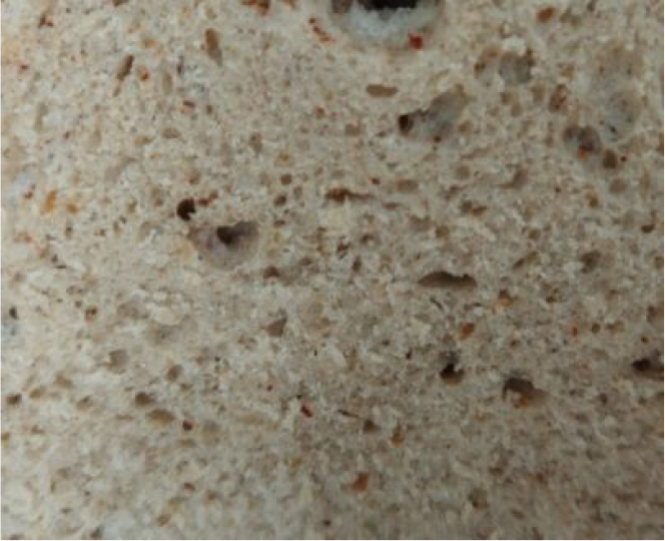	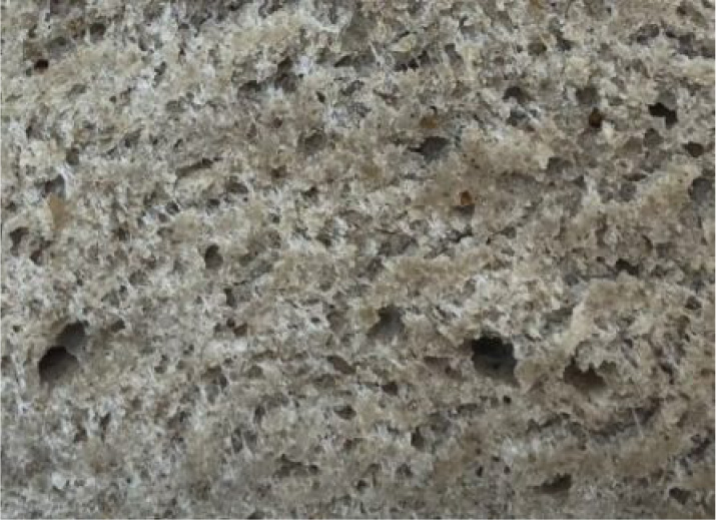
Cr‐10%	Cr‐15%

*Note*: Data expressed as mean values (*n* = 3) ± standard deviation (SD). The influence of analyzed factor (different quantities of cricket flour) on bread parameters is significant when *p* ≤ .05. Control—bread prepared without edible cricket flour; Cr‐5%, Cr‐10%, and Cr‐15%—bread samples prepared with 5%, 10%, and 15%, respectively, edible cricket flour. ^a–d^Mean values within a row with different letters are significantly different (*p* ≤ .05).

Abbreviations: L* lightness; a* redness or −a* greenness; b* yellowness or −b* blueness; NBS—National Bureau of Standards units.

Comparing bread crust color coordinates, no significant differences in L* and a* color characteristics were established between the control bread samples and slices of bread prepared with 5% edible cricket flour (on average, L* coordinates 50.2 NBS and a* coordinates 9.94 NBS). However, the highest b* coordinate was found in slices of bread prepared with 15% edible cricket flour (on average, 12.9% higher than in control bread and bread with 10% edible cricket flour, and 36.6% higher than in bread with 5% edible cricket flour). Comparing bread crumb color coordinates, the addition of edible cricket flour reduced bread crumb L* (on average, 4.86% lower than in the control for slices of bread prepared with 5% edible cricket flour, 7.66% lower with 10%, and 16.6% lower with 15%). The lowest a* and b* coordinates were found for the crumb of bread samples with 5% edible cricket flour; no significant differences in crumb b* coordinates were found between control bread and bread samples with 5% edible cricket flour. The analyzed factor (different quantities of cricket flour) had a significant influence (*p* ≤ .05) on all the analyzed bread crust and crumb color coordinates (Table 2). Also, a strong negative correlation was found between bread dough pH and bread crust a* (*r* = −.754, *p* = .005). Dough TTA showed strong positive correlations with bread crust a* and crumb L* color coordinates (*r* = .596, *p* = .041 and *r* = .660, *p* = .019, respectively), as well as a very strong negative correlation with bread crumb b* color coordinate (*r* = −.795, *p* = .002). Moderate and strong negative correlations were found between bread's specific volume and bread crust L* and b* color coordinates (*r* = −.586, *p* = .045 and *r* = .702, *p* = .011, respectively), and a strong positive correlation was established with bread crust a* color coordinate (*r* = .744, *p* = .006). Very strong correlations were found between bread porosity and all the evaluated crust and crumb color coordinates: negative correlations with crust L* and b*, as well as crumb a* and b* (*r* = −0.836, *p* = .001; *r* = −.900, *p* = .0001, *r* = −.854, *p* = .0001; *r* = −.608, *p* = .036, respectively), and positive correlations with crust a*, as well as crumb L* (*r* = .856, *p* = .0001; *r* = .803, *p* = .002). These correlations could be associated with a higher degree of water migration during the baking process, as well as more intensive Maillard reactions in the bread with higher specific volume and porosity.

Edible insects are a good source of proteins and essential amino acids (Belluco et al., 2013; Osimani et al., 2018). However, the addition of edible cricket flour to the main WB formulation led to changes in the crust color, which could be related to a higher intensity of the Maillard reaction; the latter is associated with the formation of aroma and color compounds, as well as with a higher concentration of acrylamide in the end product. The aspartic acid + asparagine content in cricket flour can be 7.52% (Osimani et al., 2018), and the latter amino acid is the main precursor for acrylamide formation. Also, it has been reported that the presence of lipids affects Maillard reaction intensity (Wang et al., 2019). From this point of view, further analysis should be performed with the aim to evaluate acrylamide formation in bread enriched with more than 5% edible cricket flour.

### 3.3 Sensory characteristics, overall acceptability, and emotions induced for consumers of bread prepared with different quantities of edible cricket flour

**FIGURE 2:**
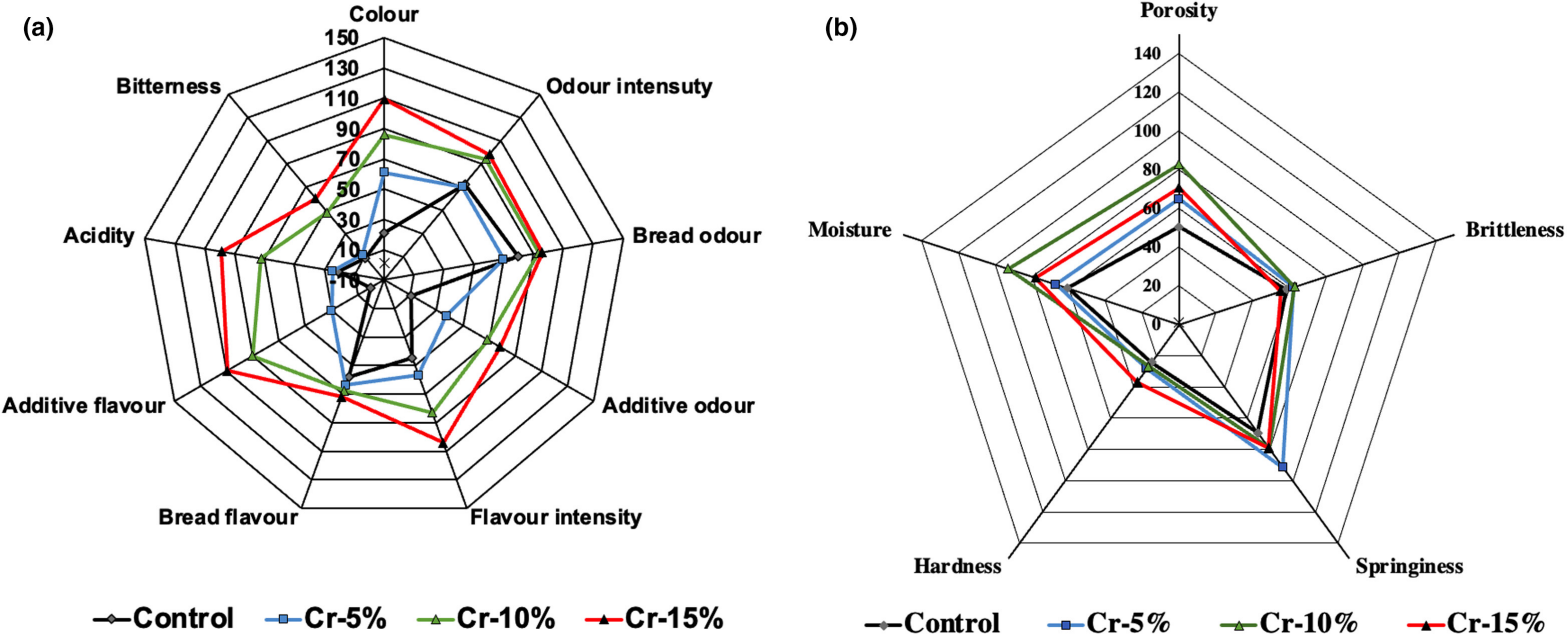
Sensory characteristic profiles (a—color, odor, and flavor characteristics; b—texture characteristics) of wheat bread prepared with and without edible cricket flour (control—bread prepared without edible cricket flour; Cr‐5%, Cr‐10%, Cr‐15%—bread prepared with 5%, 10%, and 15% edible cricket flour, respectively).

The sensory characteristic (color, odor, flavor, and texture) profiles of bread prepared with different quantities of edible cricket flour are shown in Figure 2a,b (Figure 2a—color, odor, and flavor characteristics; Figure 2b—texture characteristics). Sensory evaluation showed that the bread samples containing edible cricket flour had a significantly more intense color in comparison with control bread samples (Figure 2a). Additive odor was detected in slices of bread with 10% and 15% edible cricket flour; these slices of bread also had a higher overall flavor intensity. No significant differences between the bread's flavor were established; however, intense additive flavor was detected in slices of bread with 10% and 15% edible cricket flour. Also, slices of bread with 10% and 15% edible cricket flour showed a higher intensity of acidity and bitterness flavors, in comparison with control bread and slices of bread with 5% edible cricket flour (Figure 2a). Comparing the bread's texture sensory characteristics, no significant differences were found between the tested bread groups (Figure 2b). Bread overall acceptability and emotions induced for judges are given in Table 3. No significant differences were established between the overall acceptability of bread samples; however, some differences were found between the emotions induced for judges. A significantly higher intensity of the emotions “happy” and “sad” was expressed for slices of bread with 15% edible cricket flour (74.1% and 53.1% higher, respectively, in comparison with control bread and bread samples with 5% and 10% edible cricket flour). Also, control bread samples induced the highest intensity of the emotions “angry,” “surprised” (similar to slices of bread with 10% edible cricket flour), and “disgusted” (similar to slices of bread with 15% edible cricket flour). Very strong positive correlations were found between overall acceptability of the bread and the emotions “neutral” and “contempt” (*r* = .906, *p* = .0001 and *r* = .816, *p* = .001, respectively). Also, a very strong positive correlation was found between valence and overall acceptability (*r* = .808, *p* = .001). The analyzed factor (different quantities of cricket flour) had a significant influence on the induced emotions “happy” (*p* = .005), “sad” (*p* = .014), “surprised” (*p* = .005), and “disgusted” (*p* = .026; Table 3). Various food can induce various emotions, as well as changing emotional states (Godard et al., 2016; Macht & Dettmer, 2006). The emotions “happy” and “surprised” are more often associated with sweet foods than with salty, sour, or bitter ones, and a bitter taste is associated with the emotion “disgusted” (Macht & Mueller, 2007; Pandolfi et al., 2016; Rousmans et al., 2000). However, new, unusual, nontraditional ingredients can induce different emotions. Despite it being reported that bread is associated with a neutral emotional status (Pandolfi et al., 2016), new ingredients can increase other emotions. In this study, the emotion “happy” was expressed more intensely for bread containing edible cricket flour. This could be associated with the new experience of testing a future protein source, unusual for northeast European countries. Also, the judges were informed about the new ingredient, which could be associated with sustainable agriculture as well as with progress in reducing the problems associated with climate change.

**TABLE 3:** Overall acceptability and emotions induced for consumers by bread samples enriched with edible cricket flour

Bread samples	Overall acceptability	Emotions induced by the bread (from 0 to 1)								
		Neutral	Happy	Sad	Angry	Surprised	Scared	Disgusted	Contempt	Valence
Control	129.8 ± 20.4^a^	0.807 ± 0.167^a^	0.014 ± 0.007^a^	0.023 ± 0.009^a^	0.054 ± 0.023^c^	0.055 ± 0.019^b^	0.003 ± 0.002^a^	0.021 ± 0.009^b^	0.002 ± 0.002^a^	0.085 ± 0.035^a,b^
Cr‐5%	125.1 ± 29.7^a^	0.816 ± 0.133^a^	0.014 ± 0.009^a^	0.025 ± 0.008^a^	0.038 ± 0.014^b^	0.008 ± 0.005^a^	0.005 ± 0.003^a^	0.003 ± 0.002^a^	0.002 ± 0.002^a^	0.064 ± 0.024^a^
Cr‐10%	120.5 ± 39.0^a^	0.861 ± 0.106^a^	0.017 ± 0.008^a^	0.016 ± 0.005^a^	0.015 ± 0.008^a^	0.033 ± 0.014^b^	0.001 ± 0.001^a^	0.009 ± 0.005^a^	0.003 ± 0.003^a^	0.034 ± 0.032^a^
Cr‐15%	109.7 ± 60.7^a^	0.793 ± 0.193^a^	0.054 ± 0.017^b^	0.049 ± 0.014^b^	0.036 ± 0.011^b^	0.011 ± 0.005^a^	0.005 ± 0.003^a^	0.010 ± 0.004^a,b^	0.003 ± 0.002^a^	0.078 ± 0.053^a^

Influence of analyzed factor (different quantities of cricket flour) on bread parameters			
Bread parameters		*F*	*p*
Overall acceptability		.136	.935
Emotions induced for consumers	Neutral	.110	.952
	Happy	9.497	.005
	Sad	6.762	.014
	Angry	3.379	.075
	Surprised	9.468	.005
	Scared	1.913	.206
	Disgusted	5.357	.026
	Contempt	0.190	0.900
	Valence	1.087	.408

*Note*: Data expressed as mean values (*n* = 10) ± standard deviation (SD). The influence of analyzed factor (different quantities of cricket flour) on bread parameters is significant when *p* ≤ .05. Control—bread prepared without edible cricket flour; Cr‐5%, Cr‐10%, and Cr‐15%—bread prepared with 5%, 10%, and 15%, respectively, edible cricket flour. ^a–c^Mean values within a row with different letters are significantly different (*p* ≤ .05).

### 3.4 Volatile compound profile of bread prepared with different quantities of edible cricket flour and its correlation with overall acceptability and emotions induced for judges

The VC profile of WB prepared with different quantities of edible cricket flour and its correlation with overall acceptability and emotions induced for judges are given in Table 4. Analyzing the profile of VC, it was established that ethanol; 1‐butanol and 3‐methyl; 1‐hexanol; estragole; and hexanoic acid are the main compounds in the WB (>5% of the total VC). Comparing the percentage of these compounds in WB samples, it was found that by increasing the concentration of edible cricket flour in the main bread formula, hexanoic acid was increased; however, opposite tendencies were established for estragole: in slices of bread with edible cricket flour, the estragole content was, on average, 4.3 times lower.

**TABLE 4:** Volatile compound profile of bread samples enriched with edible cricket flour

RT, min	Volatile compounds	Bread samples				Pearson correlation (*r*) and significance (*p*)			
						*r*	*p*	*r*	*p*
		Control	Cr‐5%	Cr‐10%	Cr‐15%	OA/VC		“H”/VC	
3.18	Ethanol	14.6 ± 0.3^b^	15.5 ± 0.2^c^	9.80 ± 0.31^a^	9.50 ± 0.25^a^	.2507	.4318	−.5476	.0653
7.204	Hexanal	1.32 ± 0.19^c^	1.03 ± 0.09^c^	0.493 ± 0.029^b^	0.136 ± 0.015^a^	.3000	.3434	−.651*	**.0219**
10.088	Methanesulfonic anhydride	nd	1.68 ± 0.11	nd	nd	.0874	.7870	−.3115	.3244
10.333	1‐Butanol, 3‐methyl	8.56 ± 0.32^c^	8.99 ± 0.25^c^	6.20 ± 0.15^b^	4.98 ± 0.28^a^	.2989	.3453	−.664*	**.0185**
10.755	Furan, 2‐pentyl	0.192 ± 0.012^a^	0.176 ± 0.023^a^	nd	nd	.2338	.4646	−.5290	.0770
11.391	1‐Pentanol	1.16 ± 0.11^c^	1.05 ± 0.10^c^	0.780 ± 0.023^a^	0.889 ± 0.034^b^	.3943	.2047	−.1788	.5782
12.002	Pyrazine, methyl	1.78 ± 0.14^d^	0.680 ± 0.028^c^	0.574 ± 0.026^b^	0.099 ± 0.008^a^	.2342	.4637	−.5683	.0539
13.497	Pyrazine, 2,6‐dimethyl	0.993 ± 0.019^d^	0.527 ± 0.019^c^	0.464 ± 0.028^b^	0.393 ± 0.021^a^	.2440	.4448	−.4348	.1577
13.613	Pyrazine <2‐ethyl‐>	1.37 ± 0.12^d^	0.897 ± 0.027^c^	0.782 ± 0.019^b^	0.524 ± 0.018^a^	.2818	.3749	−.5910*	**.0431**
13.944	1‐Hexanol	12.2 ± 0.2^d^	10.9 ± 0.4^c^	9.44 ± 0.26^b^	8.02 ± 0.09^a^	.3121	.3234	−.6700*	**.0171**
14.845	Pyrazine, 2‐ethyl‐6‐methyl	1.08 ± 0.09^c^	0.499 ± 0.029^b^	0.395 ± 0.021^a^	0.357 ± 0.021^a^	.2492	.4347	−.3826	.2197
15.007	Nonanal	0.828 ± 0.017^c^	0.632 ± 0.028^b^	0.712 ± 0.029^a^	0.648 ± 0.035^a^	.4035	.1934	−.2370	.4583
15.155	Ethanol, 2‐butoxy	0.416 ± 0.016^b^	nd	nd	0.233 ± 0.018^a^	.0760	.8144	.1955	.5425
15.337	Pyrazine, 2‐ethyl‐3‐methyl	1.96 ± 0.08^d^	0.905 ± 0.035^c^	0.785 ± 0.018^b^	0.608 ± 0.021^b^	.2187	.4946	−.4484	.1438
15.615	1,3‐Hexadiene, 3‐ethyl‐2‐methyl	0.698 ± 0.022^d^	0.413 ± 0.024^c^	0.221 ± 0.015^a^	0.266 ± 0.019^b^	.2459	.4410	−.3719	.2340
16.102	1‐Octen‐3‐ol	1.45 ± 0.13^d^	1.01 ± 0.09^c^	0.811 ± 0.020^b^	0.686 ± 0.023^a^	.3152	.3183	−.4892	.1065
16.248	1‐Heptanol	1.19 ± 0.11^b^	0.602 ± 0.036^a^	0.552 ± 0.014^a^	0.583 ± 0.013^a^	.2301	.4719	−.2681	.3994
16.866	Furfural	2.70 ± 0.18^a^	2.58 ± 0.24^a^	8.37 ± 0.16^c^	5.30 ± 0.21^b^	−.0472	.8842	.2144	.5034
17.071	2‐Propanol, 1‐(2‐methoxy‐1‐methylethoxy)	nd	nd	0.257 ± 0.011	nd	.0058	.9858	−.2264	.4791
17.801	Ethanone, 1‐(2‐furanyl)	nd	0.827 ± 0.025^a^	1.17 ± 0.09^b^	0.843 ± 0.024^a^	−.0631	.8456	.2285	.4749
17.989	2‐Propanol, 1‐(2‐methoxypropoxy)	nd	nd	0.650 ± 0.030	nd	.0073	.9821	−.2259	.4803
18.268	Benzaldehyde	0.990 ± 0.025^a^	1.49 ± 0.08^b^	1.85 ± 0.15^c^	2.26 ± 0.19^d^	.0009	.9978	.7810**	.**0027**
18.361	2‐Nonenal, (E)	0.748 ± 0.014^b,c^	0.700 ± 0.031^b^	0.660 ± 0.018^b^	0.454 ± 0.025^a^	.3631	.2460	−.7770**	**.0029**
18.681	1‐Octanol	0.717 ± 0.032^d^	0.270 ± 0.024^a^	0.362 ± 0.023^b^	0.489 ± 0.029^c^	.1701	.5972	.1258	.6968
18.964	Propanoic acid, 2‐methyl	nd	0.181 ± 0.012^a^	0.214 ± 0.019^a^	0.210 ± 0.018^a^	−.0370	.9092	.4273	.1659
19.192	3,5‐Octadien‐2‐one	0.648 ± 0.028^c^	0.630 ± 0.023^c^	0.554 ± 0.017^b^	0.492 ± 0.023^a^	.4571	.1352	−.6050*	**.0370**
19.307	2‐Furancarboxaldehyde, 5‐methyl	0.870 ± 0.036^b^	0.762 ± 0.031^a^	2.64 ± 0.21^d^	1.78 ± 0.12^c^	−.0564	.8618	.2716	.3932
19.449	Hexadecane	0.168 ± 0.023^a^	0.183 ± 0.015^a^	nd	0.191 ± 0.011^a^	.0909	.7789	.3501	.2646
20.838	1‐Nonanol	1.01 ± 0.07^d^	0.750 ± 0.020^b^	0.254 ± 0.023^a^	0.941 ± 0.028^c^	.0864	.7896	.3299	.2950
20.99	3‐Furanmethanol	3.85 ± 0.19^d^	3.07 ± 0.29^c^	2.46 ± 0.18^b^	1.66 ± 0.09^a^	.3464	.2700	−.6540*	**.0211**
21.253	Estragole	8.54 ± 0.14^d^	0.505 ± 0.014^a^	1.10 ± 0.09^b^	4.35 ± 0.14^c^	.0758	.8149	.0976	.7628
21.377	3‐Nonen‐1‐ol, (Z)	0.364 ± 0.028^d^	0.302 ± 0.026^c^	0.162 ± 0.011^a^	0.201 ± 0.015^b^	.3250	.3026	−.3218	.3077
21.646	Alpha‐terpineol	0.231 ± 0.011^b^	nd	nd	0.109 ± 0.009^a^	.0947	.7696	.1173	.7166
22.503	Pentanoic acid	0.766 ± 0.023^a^	0.758 ± 0.033^a^	0.989 ± 0.036^b^	1.11 ± 0.12^b^	.1196	.7112	.8410**	**.0006**
22.611	D‐Carvone	0.492 ± 0.014^c^	nd	0.212 ± 0.015^a^	0.259 ± 0.014^b^	.0922	.7758	.0706	.8274
22.969	Benzenemethanol, alpha‐methyl‐alpha‐(1‐methyl‐2‐propenyl)	0.227 ± 0.015	nd	nd	nd	.1609	.6175	−.3140	.3203
23.45	Propanoic acid, 2‐methyl‐, phenylmethyl ester	0.290 ± 0.013	nd	nd	nd	.1561	.6281	−.3173	.3149
23.588	Dec‐(4Z)‐en‐1‐ol	nd	nd	0.192 ± 0.012^a^	0.469 ± 0.024^b^	−.1653	.6077	.8520**	**.0004**
23.625	Ethanol, 1‐(2‐butoxyethoxy)	0.351 ± 0.012	nd	nd	nd	.1537	.6335	−.3189	.3123
24.100	2,4‐Decadienal, (E,E)	0.230 ± 0.009^b^	0.302 ± 0.021^d^	0.263 ± 0.019^c^	0.199 ± 0.013^a^	.3961	.2024	−.4894	.1063
24.417	1H‐Pyrrole, 1‐(2‐furanylmethyl)	0.157 ± 0.008^b^	0.112 ± 0.011^a^	0.162 ± 0.011^b^	0.186 ± 0.015^b^	.1913	.5515	.7490**	**.0051**
24.558	Hexanoic acid	10.9 ± 0.1^a^	11.3 ± 0.4^a^	14.5 ± 0.3^b^	15.6 ± 0.3^c^	−.0815	.8012	.7330**	**.0067**
24.746	2‐Cyclohexen‐1‐one	nd	21.6 ± 0.3^a^	23.5 ± 0.5^b^	24.7 ± 0.6^c^	−.1320	.6827	.4080	.1880
25.022	Propanoic acid, 2‐methyl‐, 3‐hydroxy‐2,4,4‐trimethylpentyl ester	4.43 ± 0.15^d^	0.418 ± 0.036^a^	0.630 ± 0.032^b^	1.86 ± 0.14^c^	.1232	.7028	.0007	.9984
25.361	Propanoic acid, 2‐methyl‐, 2,2‐dimethyl‐1‐(2‐hydroxy‐1‐methylethyl)propyl ester	4.02 ± 0.23^d^	0.441 ± 0.027^a^	0.625 ± 0.029^b^	1.86 ± 0.13^c^	.1223	.7051	.0407	.9000
25.981	Phenylethyl alcohol	2.03 ± 0.18^c^	1.94 ± 0.08^b^	1.32 ± 0.12^a^	1.10 ± 0.12^a^	.4214	.1725	−.5492	.0644
26.535	Heptanoic acid	0.903 ± 0.028^b^	0.836 ± 0.029^a^	1.05 ± 0.09^c^	1.16 ± 0.14^c^	.2695	.3970	.8460**	**.0005**
27.061	Maltol	nd	0.723 ± 0.031	nd	nd	.0799	.8051	−.3159	.3172
28.208	2(3H)‐Furanone, dihydro‐5‐pentyl	1.87 ± 0.16^a^	1.90 ± 0.09^a^	1.82 ± 0.15^a^	1.83 ± 0.23^a^	.950**	**.0001**	.3378	.2829
28.411	Octanoic acid	1.29 ± 0.09^c^	0.644 ± 0.023^a^	0.890 ± 0.056^b^	1.17 ± 0.11^c^	.1951	.5435	.4356	.1570
30.075	Ethanol, 2‐phenoxy	0.120 ± 0.014^a^	nd	nd	0.141 ± 0.012^a^	.0075	.9815	.5990*	**.0395**
30.205	Nonanoic acid	0.704 ± 0.017^a^	0.691 ± 0.035^a^	0.692 ± 0.029^a^	0.829 ± 0.029^b^	.1574	.6252	.9740**	**.0001**
30.991	Guaiacol <4‐vinyl‐>	0.590 ± 0.019^c^	0.592 ± 0.026^c^	0.452 ± 0.025^b^	0.325 ± 0.017^a^	.3493	.2657	−.7130**	**.0092**

*Note*: Data expressed as mean values (*n* = 3) ± standard deviation (SD). ^a–d^Mean values within a column with different letters are significantly different (*p* ≤ .05). Pearson correlation is significant when *p* ≤ .05. ^*^ indicates correlation is significant at the 0.05 level (2‐tailed), ^*^^*^ indicates correlation is significant at the 0.01 level (2‐tailed).

Abbreviations: “H”, emotion “happy” fixed by FaceReader; Control, bread prepared without edible cricket flour; Cr‐5%, Cr‐10%, and Cr‐15%, bread prepared with 5%, 10%, and 15%, respectively, edible cricket flour; nd, not determined; OA, overall acceptability; r, Pearson correlation coefficient; RT, retention time; VC, volatile compound.

Similar tendencies were found for 1‐butanol, 3‐methyl‐, and 1‐hexanol: on increasing the percentage of edible cricket flour, the concentration of 1‐butanol, 3‐methyl, and 1‐hexanol was reduced. However, the highest concentration of ethanol was established in slices of bread with 5% edible cricket flour (15.5% of the whole VC). Also, strong negative correlations were found between 1‐butanol and 1‐hexanol and the emotion “happy” induced for judges (*r* = *−*.664, *p* = .0185 and *r* = .6700, *p* = .0171, respectively). In contrast to the above‐mentioned findings, a strong positive correlation was established between the emotion “happy” and hexanoic acid (*r* = .7330, *p* = .0067). Ethanol odor is characterized as vinous; 1‐butanol odor is described as fusel, oily, sweet, balsamic, and whiskey; 3‐methyl odor as fusel, alcoholic, pungent, etherical, cognac, fruity, banana, and molasses; 1‐hexanol odor as pungent, ethereal, fusel oil, fruity and alcoholic, and sweet with a green top note; estragole odor as sweet, phenolic, anise, harsh, spice, green, herbal, and minty; and hexanoic acid odor as cheesy, fruity, phenolic, fatty, and goaty.

Despite the fact that some compounds were found in smaller amounts in the bread VC profile, they showed a significant correlation (positive and/or negative) with the emotion “happy” expressed in the faces of judges. Strong negative correlations were found between emotion “happy” and hexanal (*r* = *−*.6510, *p* = .0219), pyrazine <2‐ethyl‐> (*r* = *−*.5910, *p* = .0431), 2‐nonenal, (E)‐ (*r* = *−*.7770, *p* = .0029), 3,5‐octadien‐2‐one (*r* = *−*.6050, *p* = .0370), 3‐furanmethanol (*r* = *−*.6540, *p* = .0211), and guaiacol <4‐vinyl‐> (*r* = *−*.7130, *p* = .0092). The content of most of the above‐mentioned VC was lower in slices of bread prepared with edible cricket flour, in comparison with control bread samples (except 3,5‐octadien‐2‐one and guaiacol <4‐vinyl‐>, the content of which was similar in control bread and samples prepared with 5% edible cricket flour). Hexanal odor is characterized as fresh, green, fatty, aldehydic, grass, leafy, fruity, and sweaty; pyrazine<2‐ethyl‐> odor is described as peanut butter, musty, nutty, woody, and roasted cocoa; 2‐nonenal, (E)‐ odor as fatty, green, cucumber, aldehydic, and citrus; 3,5‐octadien‐2‐one odor as fruity, fatty, and mushroom; 3‐furanmethanol is described as a bready‐type odor; and guaiacol <4‐vinyl‐> odor is associated with dry, woody, fresh, amber, cedar, roasted, and peanut.

Positive correlations were found between the emotion “happy” and benzaldehyde (*r* = .7810, *p* = .0027), pentanoic acid (*r* = .8410, *p* = .0006), dec‐(4Z)‐en‐1‐ol (*r* = .8520, *p* = .0004), 1H‐pyrrole, 1‐(2‐furanylmethyl) (*r* = .7490, *p* = .0051), and heptanoic acid (*r* = .8460, *p* = .0005), ethanol, 2‐phenoxy‐ (*r* = .5990, *p* = .0395), and nonanoic acid (*r* = .9740, *p* = .0001). Comparing the above‐mentioned VC in bread samples, the lowest content of benzaldehyde was found in control bread samples (0.990% of the total VC), and on increasing the edible cricket flour content in the main bread formula, benzaldehyde was increased. A similar pentanoic acid content was established in control bread and bread prepared with 5% edible cricket flour (on average, 0.762% of the total VC); however, on increasing the edible cricket flour content in the main bread formula, pentanoic acid was increased. The 1H‐pyrrole, 1‐(2‐furanylmethyl) content in control bread and bread samples with 10% and 15% edible cricket flour was similar, and the lowest content of 1H‐pyrrole, 1‐(2‐furanylmethyl) was found in bread samples prepared with 5% edible cricket flour. The highest heptanoic acid content was established in slices of bread with 10% and 15% edible cricket flour (on average, 1.11% of the total VC), and the highest nonanoic acid content was found in bread samples prepared with 15% edible cricket flour (0.829% of the total VC). Benzaldehyde odor is described as strong, sharp, sweet, bitter, almond, and cherry; pentanoic acid odor as acidic and sharp, cheese‐like, sour milky, tobacco, with fruity nuances; dec‐(4Z)‐en‐1‐ol odor as waxy, fatty, and fruity; 1H‐pyrrole, 1‐(2‐furanylmethyl) odor is associated with plastic, green, waxy, fruity, coffee, and vegetable; heptanoic acid odor as cheesy, waxy, sweaty, fermented, pineapple, and fruity; ethanol, 2‐phenoxy odor is described as mild, rose, balsam, and cinnamyl; and nonanoic acid odor as waxy, dirty, and cheesy with a cultured dairy nuance.

Bread aroma is one of the first quality parameters perceived by consumers and a most important characteristic of product acceptance. However, sensory analysis methods are limited by human perception (Hansen & Schieberle, 2005; Pico et al., 2016). Our experiment showed that despite the results of overall acceptability of bread not being correlated with the VC profile, significant correlations were established with the emotion “happy” and separate VC. More than 540 VC can be formed in bread but only a relatively small number of those contribute to desirable aroma properties (Cho & Peterson, 2010; Pico et al., 2016). Bread VC profile is related to formula ingredients, enzymatic reactions (during fermentation processes), and thermal reactions (during baking; Kirchhoff & Schieberle, 2001). Our study showed that the analyzed factor (different quantities of edible cricket flour) had a significant effect (*p ≤* .05) on most of the VC identified in bread (except 2[3H]‐furanone and dihydro‐5‐pentyl). Finally, our study is in agreement with previous findings that VC formation is related to many factors, and despite the overall acceptability of the different slices of bread being similar, different VC could be related to the emotions induced for consumers.

## 4 CONCLUSIONS

The influence of edible cricket flour on dough and bread parameters varies and, in most cases, is related to the quantity of additives. Edible cricket flour reduces dough pH, redness, and yellowness. However, 5% edible cricket flour increases the porosity of bread by 7.87%. Sensory analysis showed that the edible cricket flour led to a more intense bread color and additive odor. Also, slices of bread with 10% and 15% edible cricket flour showed a higher intensity of overall, additives, acidity, and bitterness flavors. However, all bread samples showed similar overall acceptability and no correlations were found between bread overall acceptability and VC. However, a higher intensity of the emotions “happy” and “sad” was expressed for bread samples prepared with 15% insect flour, and significant correlations were established between the emotion “happy” and separate VC. Finally, 5% edible cricket flour could be incorporated into the main WB formula without a negative impact on bread quality. Also, edible cricket flour influences the formation of VC, and separate VC could be related to emotions induced for consumers.

## CONFLICT OF INTEREST

The authors declare no conflict of interest.

## Data Availability

The data are available from the corresponding author, upon reasonable request.
